# Hydrodynamic properties of macromolecules and nanoparticles in dilute solution: a brief essay on classical and modern concepts

**DOI:** 10.1007/s00249-025-01791-7

**Published:** 2025-08-24

**Authors:** José García de la Torre, José G. Hernández-Cifre

**Affiliations:** Departamento de Química Física, Universidad de Murcia, Campus de Espinardo, 30100 Murcia, Spain

**Keywords:** Hydrodynamic theory/methods, Computational modelling, Equivalent radii, Analytical Ultracentrifugation

## Abstract

The theory, computational modelling and data analysis of hydrodynamic and other solution properties of macromolecules and nanoparticles in dilute solution are nowadays well-established. Along this essay, we briefly present the variety of methods which are currently available for those purposes. Although such methods embody an important complexity, they are usually presented as user-friendly tools which can be used without previous knowledge of their foundations. Some understanding of classical concepts in which modern tools are based can result in a better, more profitable, use of them and a most adequate form of presenting and discussing their results. We describe the utility of employing a systematic way of handling data and results for the solution properties in terms of equivalent radii, which indeed provide an alternative to the raw properties in their use for structural determinations. They can also be employed in the design of simulation of experiments and data analysis procedures, like in analytical ultracentrifugation as we propose finally in this paper.

## Introduction

It is now over 100 years, since the impressive contribution of T. Svedberg, namely, the invention of the analytical ultracentrifugation (AUC) (Cölfen 2023), settled down the concepts advanced 120 years ago by Einstein (1905). The theories of Einstein, based on *simple* properties of common substances—e.g., sucrose—in dilute aqueous solution, would provide evidence of the molecular nature of matter. And then, Svedberg and Nichols (1923), Svedberg and Pedersen (1940) provided the evidence of the existence of *macro-molecules*. The concepts exposed by Einstein are still the basis of the study of macromolecules in solution, and the principles of Svedberg have pervaded through the yeas in modern AUC instrumentation and data analysis.

A look at the present status of methodologies for the characterization of macromolecules (and more recently, nanoparticles) shows that the enormous advances reached in theoretical and instrumental aspects still employ terms and concepts based on those venerable works. On the theoretical side, the implementation of those concepts in computational tools has been made keeping in mind the obtention of experimentally observable properties; for instance, the diffusion coefficient, *D*, the sedimentation coefficient, *s*, or the intrinsic viscosity, $[\eta ]$. It is somehow overlooked that hydrodynamic theories predict just an effective hydrodynamic radius or volume. The comparison of hydrodynamic calculation vs. experimental values (or between the outcome of different computational models) could be made in terms of them, rather than comparing values of the experimental properties, *D*, *s*, $[\eta ]$, etc. We shall describe below some advantages of such approach. The extraction of structural properties (size, shape, conformation, etc) from experimental data, or their computational prediction, is often based on properties which, like *s* and $[\eta ]$, depend not only on the overall structure of the particle, but also on its molecular mass, and other non-structural properties of the solute and the solvent.

Other aspects evidence an adherence, in data treatment or reporting, to classical forms of combining properties—like the frictional ratio, $f/f_0$, or the ratio of the radius of gyration to the hydrodynamic radius, $R_g/R_H$ (definitions and notations are indicated below)—which could be replaced by more systematic combinations. Such points of view about formal aspects, along with a summary of the various modelling, theoretical and computational approaches, available for rigid or flexible biomacromolecules and synthetic polymers, are considered in this essay.

## The classical theories

The foundations of the present knowledge on hydrodynamics of macromolecules and nanoparticles in dilute solution were established in the seminal work of Einstein (1905, 1906, 1911). It seems convenient to recall some aspects of these theories that, apart from his famous equations, contain essential concepts.

Einstein had the great insight of relating the diffusion coefficient of a particle, *D*, to the frictional coefficient, *f*, which is related to the force, $F=fv$, that a particle experiences when it moves with velocity *v* in low-Reynolds-number conditions (as it happens for microscopic particles in ordinary solvents; see Appendix). Classical fluid mechanics tell us that, in such conditions, *f* will always be proportional to the solvent viscosity, $\eta _0$, so that it can be expressed as

1 $$\begin{aligned} f = f^* \eta _0 \end{aligned}$$

where the reduced friction coefficient, $f^*$, is a quantity that just depends on the particle’s geometry, i.e., on its size and shape. The Stokes law states that for a spherical particle of radius *R*, $f^*=6 \pi R$. Combining fluid mechanics with thermodynamics, Einstein arrived at his famous equation, $D = k_BT/f$, where $k_B$ is the Boltzmann’s constant and *T* is the absolute temperature. This expression holds for any particles of any kind. For rigid, spherical particles:

2 $$\begin{aligned} D = \frac{k_BT}{6 \pi \eta _0 R} \end{aligned}$$

In general, for rigid particles, the reduced friction coefficient can be factored out into two terms:

3 $$\begin{aligned} f^* = f^*_{shape} f^*_{size} \end{aligned}$$

one depending on size, and another depending just on shape. For the spherical particle, $f^*_{shape} = 6 \pi$ and $f^*_{size} = R$.

In a subsequent paper (Einstein 1906), Einstein developed the theory of solution viscosity. His main, general result is that for rigid solute particles, the relative viscosity—ratio of the viscosity of the solution, $\eta$, to that of the solvent—is given by a very simple, linear relationship involving the volume fraction of the solute, $\phi$:

4 $$\begin{aligned} \eta _{rel} \equiv \eta / \eta _0 = 1 + \nu \phi \end{aligned}$$

where $\nu$ is a numerical, dimensionless factor which depends only on the shape of the particle, but not on its size (two particles with same shape and different size are isomorphic to each other). For spherical particles, Einstein was able to obtain $\nu _p = 5/2$. Nowadays, the quantity that characterizes the increment in viscosity due to the solute over that of the solvent is the intrinsic viscosity, $[\eta ]=(\eta _{rel}-1)/c$, where *c* is the mass concentration. The ratio of volume fraction to mass concentration is determined by the volume, $V_p$, and mass, $m=M/N_A$, of the particles, such that

5 $$\begin{aligned} {[}\eta ] = \nu \frac{N_A V_p}{M} \end{aligned}$$

where *M* is the molecular weight of the particles and $N_A$ is the Avogadro’s number. A full description of these aspects is presented in the Appendix, where we show that $[\eta ]$ depends, in addition to the molecular mass, solely on a hydrodynamic effective volume $V_H=(2/5)\nu _p V_p$. For a spherical particle of radius, *R*, $\nu _p =5/2$, so that the effective volume is that of the sphere, $V_p=4\pi R^3/3$ and then:

6 $$\begin{aligned} {[}\eta ] = \frac{10 \pi N_A R^3}{3M} \end{aligned}$$

Further details about theoretical aspects for the intrinsic viscosity can be found elsewhere (García de la Torre and Carrasco 1998; García de la Torre et al. 2007).

An essential sequel of the theories of Einstein was the invention of Svedberg of the analytical ultracentrifugation (AUC) (Svedberg and Nichols 1923). This topic is treated in some detail in a later section. The sedimentation coefficient, *s*, which is the most immediate property measured by this technique, is directly related to the frictional coefficient:

7 $$\begin{aligned} s = \frac{M(1-{\bar{v}} \rho )}{N_A f} \end{aligned}$$

where $M(1-{\bar{v}} \rho ) \equiv M^{(b)}$ is the buoyant molecular weight, which includes the buoyancy factor $1-{\bar{v}} \rho$, where ${\bar{v}}$ is the partial specific volume of the solute particles and $\rho$ is the solution density, nearly equal to the solvent density for dilute solution.

The next landmarks in macromolecular hydrodynamics were the obtention of exact, analytical expressions for the hydrodynamic properties of revolution ellipsoids with semiaxes *a*, *b* and *b* (i.e., with two equal semiaxes), and axial ratio $p=a/b$. This simple model provided a way to analyze experimental properties of non-spherical particles, with elongated or flattened shapes, in terms of prolate ellipsoids with $p>1$ or oblate ellipsoids with $p<1$. Perrin (1934) derived the theoretical result for the friction coefficient, and Simha (1940) found the results for the viscosity. Particularly, the Perrin’s theory complies with what is expressed in Eqs. (1) and (3); his equation for *f* can be recast into the form:

8 $$\begin{aligned} f = \eta _0 (ab^2)^{1/3} 6\pi P(p) \end{aligned}$$

The volume of the ellipsoid is $V = 4 \pi a b^2/3$, and *P*(*p*), sometimes called Perrin function, is a function of the axial ratio. Clearly, *f* is of the above indicated form, with $f^*_{size}=(3V)^{1/3}=(ab^2)^{1/3}$, i.e., the geometric mean of the three semiaxes, and $f^*_{shape}= 6 \pi P(p)$, which depends only on the shape of the ellipsoid expressed by the axial ratio *p*. The primary result was an analytical, explicit formula for the *P*(*p*) function [a correction to the Perrin’s results was reported by Koening (1975)].

For the viscosity, Simha obtained a result of the form of the Einstein equation (4), or its equivalent equation (5) for $[\eta ]$, with the main result being an exact, explicit expression for the Einstein viscosity coefficient $\nu (p)$.

## The frictional ratio, $f/f_0$

The theories for hydrodynamic properties of ellipsoidal particles were immediately accepted in the then nascent field of macromolecular science, and particularly in molecular biology, to gather information about the overall shape of biomolecules; for instance, to distinguish between globular and fibrous proteins with basis on diffusion, sedimentation and viscosity measurements. It was noticed that the theoretical result of Perrin could be presented as the ratio of the frictional coefficient of the particle to the frictional coefficient of a sphere having the same volume as the particle. The radius of this equivalent sphere is $[3V_p/(4\pi )]^{1/3}$, and its frictional coefficient is then

9 $$\begin{aligned} f_0 = 6 \pi \eta _0 \biggl ( \frac{3V_p}{4\pi } \biggr )^{1/3} \end{aligned}$$

Therefore, the ratio $f/f_0$ depends only on the shape of the ellipsoid, given by its axial ratio *p*, but not on its actual size. Indeed, connecting this definition with the formalism presented in our previous section, one immediately finds that the frictional ratio is the same as the Perrin function, $f/f_0 \equiv P(p)$.

For the elucidation of the overall shape of rigid particles from experimental data of *s* or *D* coefficients, they could be combined with the molecular weight (obtained, for instance, from Svedberg’s equation for the *s*/*D* ratio). Such combination is expressed by

10 $$\begin{aligned} f/f_0 = \frac{f}{ 6\pi \eta _0} \biggl ( \frac{4 \pi N_A}{3M({\bar{v}})'} \biggr )^{1/3} \end{aligned}$$

where $({\bar{v}})'$ is the partial specific volume of the particle including hydration as explained below. Then, from the experimentally obtained value of $f/f_0$ one could estimate the axial ratio.

This procedure was applied for a long time to proteins—perhaps just to distinguish fibrous from globular ones—and other biomacromolecules. In such cases, an important aspect is hydration, which increases the volume extracted from hydrodynamic measurements (Tanford 1961; Van Holde et al. 1998; García de la Torre 2001b). Hydration was taken into account in a somehow primitive manner, assuming a uniform expansion of the anhydrous particle given as the degree of hydration, $\delta$, i.e., the grams of water per gram of the particle. Thus, the partial specific volume is increased by a factor $1+\delta /\rho$, such that the value to be used in Eq. (9) should be $({\bar{v}})'={\bar{v}}(1+\delta /\rho )$ instead of ${\bar{v}}$. Hydration is an important, and somehow controversial, problem in biophysics; we leave this topic apart as it would deserve a separate review.

## Rigid particles of arbitrary shape

The revolution-ellipsoid model just gives an indication of the overall aspect of a rigid particle, but it is certainly insufficient to describe details of the shape that are particularly important in molecular biology, as they are responsible for important functions of biomacromolecules. In the second half of last century, the advent of computers made it possible to consider such details in the analysis of hydrodynamic and other solution properties.

The description of the hydrodynamics of rigid particle begins with some generalities about how the frictional resistance and the Brownian diffusivity are expressed and related to each other. Both translational and rotational motion have to be jointly treated. Starting from the concepts developed by Brenner (1967) about translation–rotation coupling and hydrodynamic centers, successive developments have settled down this aspect (García Bernal and García de la Torre 1980; Harvey and García de la Torre 1980; Carrasco and García de la Torre 1999a, b). A summary of the theory is presented in the Appendix.

The prediction of hydrodynamic properties is made by applying such formalism to a specific model of the rigid particles. In some cases, simple models such as cylinders or ellipsoids are sufficient or acceptable, and some exact results are available for them, as mentioned above for ellipsoids. Inspired in the necklace, chain-of-beads model of flexible polymers (*vide infra*), Bloomfield et al. (1967), García de la Torre and Bloomfield (1977a), García de la Torre and Bloomfield (1981) put forward the use of bead models for the hydrodynamics of rigid particles that could be treated computationally. Advances in hydrodynamic theories, and the availability of more and more powerful computers made it possible to refine the detail of such models (García de la Torre 1989), implemented years ago in the HYDRO program (García de la Torre et al. 1994), the first one in what is now the HYDRO suite (García de la Torre 2016; García de la Torre et al. 2018b), which includes the new HYDRO++ (García de la Torre et al. 2007) along with several other programs based on the bead-model methodology, namely: HYDROPRO (García de la Torre et al. 2000a; Ortega et al. 2011b), HYDRONMR (García de la Torre et al. 2000b), HYDROMIC (García de la Torre et al. 2001), HYDROSUB (García de la Torre and Carrasco 2002), HYDROPIX (García de la Torre 2001a), and HYDRO2D (Hernández Cifre et al. 2022).

Bead models and other similar approaches are customarily used to gather structural information from hydrodynamic properties of rigid particles assemblies. Regarding biophysical applications, the methods to relate hydrodynamic properties to structural information available from high-resolution X-ray crystallography and nuclear magnetic resonance have been particularly remarkable. Among these programs are: AtoB (Byron 1997), BEAMS (Spotorno et al. 1997), HYDROPRO (García de la Torre et al. 2000a; Ortega et al. 2011b), HYDRONMR (García de la Torre et al. 2000b), ZENO (Kang et al. 2004), SOMO (Rai et al. 2005), BEST (Aragon and Hahn 2006; Aragon 2016), US-SOMO (Brookes et al. 2010b; Brookes and Rocco 2016), HullRad (Fleming and Fleming 2018), GRPY (Zuk et al. 2018), and FiniteElements (Sedeh et al. 2018). Some comparative studies of these programs are available (Rocco and Byron 2015c, a, b).

**Fig. 1:**
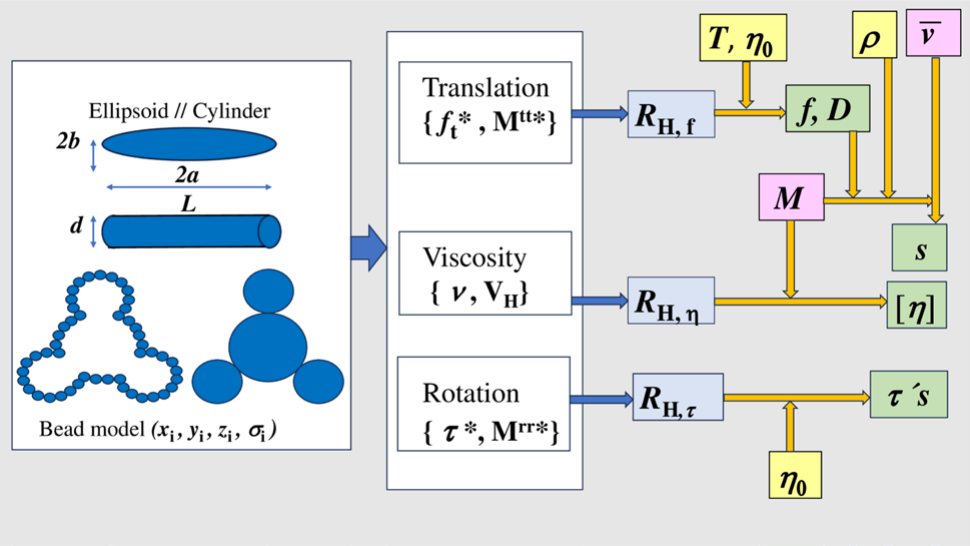
Diagram of the theoretical and computational scheme for the calculation of hydrodynamic properties. White blocks indicate the properly hydrodynamic theoretical/computational steps that finally conduct to the equivalent hydrodynamic radii (blue blocks). The obtention of experimental properties (green blocks) requires diverse quantities related to the solvent (yellow blocks) and the nature of the solute (pink blocks)

The present AlphaFold revolution (https://alphafold.ebi.ac.uk/) allows a reliable prediction of the atomic-level structures of nearly every protein, providing the coordinates from which experimental properties can be predicted; for instance, small-angle X-ray-scattering related quantities (Brookes et al. 2023) such as the radius of gyration, *Rg*. Likewise, as indicated below (Fig. 1), atomic coordinates are all one needs to run a bead-model calculation of hydrodynamic radii. This possibility opens a new avenue in macromolecular hydrodynamics, in which measurement of diffusion, sedimentation and viscosity would provide data needed for confirmation of AlphaFold structures.

Figure 1 presents a scheme showing the fundamental aspects for the calculation of hydrodynamic properties. The starting point is the geometry of a hydrodynamic model for the particle. As mentioned above, it could be a simple model such as ellipsoids and cylinders (rods or disks) whose size is given by a few parameters.

For an arbitrarily complex shape, in bead-modelling the whole geometry (size and shape) of the model is described by a set of Cartesian coordinates and radii of the beads. It is important to remark that this geometrical information (shape and parameters or coordinates) is all the primary information required to compute hydrodynamic quantities, such as mobility matrices and a hydrodynamic volume, and from them reduced hydrodynamic coefficients and, particularly hydrodynamic radii are calculated. At this point the role of hydrodynamic theory and computation is finished.

Then, the obtention of experimentally observable properties require further information on physico-chemical properties of the solute particles and the solvent: the temperature and solvent viscosity are needed for the diffusion coefficient; the solvent density, specific volume and molecular mass of the solute are needed for the sedimentation coefficient, and the molecular mass is needed for the intrinsic viscosity.

## Flexible macromolecules

The hydrodynamics of flexible particles is even more complex than that for rigid particles. Because of their flexibility, non-rigid macromolecules do not have a defined size and shape; instead, they can adopt multiple conformations. The conformational variability among the individual molecules in a sample is not just a static aspect. Furthermore, each individual molecule is continuously, dynamically changing its shape while experiencing Brownian motion.

The earliest theories and models [string of beads (Kirkwood and Riseman 1948; Kirkwood 1954) or bead-and-spring chains (Zimm 1956)] treated both hydrodynamics and conformational aspects in a rather approximate manner. Improvements in hydrodynamic theory and computational resources brought the so-called Monte Carlo rigid-body (MCRB) simulation (Zimm 1980; García de la Torre et al. 1982), in which hydrodynamic properties are calculated averaging values calculated by an instantaneous conformation, using rigid-body hydrodynamics like that for rigid particles. The MCRB method has been applied to a number of studies on biomacromolecular hydrodynamics (Iniesta and García de la Torre 1987; García de la Torre 1994; García de la Torre et al. 2003; Amorós et al. 2011), as it is fully valid for global, single-valued hydrodynamic properties, such as *s*, *D* and $[\eta ]$. A most rigorous, but costly approach, is Brownian dynamics (BD) simulation (Ermak and McCammon 1978; Iniesta and García de la Torre 1990), which is the choice when the subject is the simulation of effects related to the internal dynamics of the macromolecule (Rey et al. 1992). We have developed a software package, SIMUFLEX (García de la Torre et al. 2009), which includes both MCRB and BD simulations (García de la Torre et al. 2010) with a force field particularly adapted to coarse-grained models of biomacromolecules; indeed, BD methods are remarkably useful for simulations of solution properties and dynamics of disordered proteins (Amorós et al. 2013; Ahn et al. 2022).

## Size/shape-dependent quantities

### Equivalent radii and ratios of radii

The values, either experimental or computationally predicted, of translational friction, diffusion or sedimentation coefficients are sometimes (not frequently) expressed as a hydrodynamic radius, evaluated as the radius a sphere having the same value of *f*, *D* or *s*, obtained as

11 $$\begin{aligned} a_T \equiv \frac{f}{6 \pi \eta _0}= & \frac{k_B T}{6 \pi \eta _0 D} \nonumber \\= & \frac{M(1-{\bar{v}} \rho )}{6 \pi \eta _0 N_A s} \equiv R_{H,f} \end{aligned}$$

The usual notation for the hydrodynamic radius is $R_H$. We use $R_{H,f}$ to indicate that it comes from the Stokes–Einstein equations for these properties. It should be clear that Eq. (11) is just *a definition* of a quantity that can be derived from a value of *s* or *D*, either measured or calculated, and for any particle, rigid or flexible, of any shape or conformation.

From Eqs. (1) and (3), it comes that this hydrodynamic radius is just equal to what we call the reduced friction coefficient times a numerical factor: $a_T \equiv R_{H,f} = f^*/6\pi = 0.053 f^*$, $f^*= 18.8 R_{H,f}$.

With the same concept as for *f*, *s* and *D*, a hydrodynamic radius $R_{H, \eta }$ can be defined from Eq. (5) as the radius of a sphere having the same intrinsic viscosity (and molecular weight) as the particle that is being considered:

12 $$\begin{aligned} a_I \equiv \biggl ( \frac{3 M [\eta ]}{10 \pi N_A} \biggr )^{1/3} \equiv R_{H, \eta } \end{aligned}$$

In the Appendix, we summarize the steps in the calculation of the two radii.

Generalizing the concept of hydrodynamic radii, *equivalent radii* can be defined for other properties of the solute particles. Thus, $a_X$ would be the radius of a spherical particle having the same value of the property *X* as the solute particle. In that way, we have introduced the alternative notations $a_T$ and $a_I$ for $R_{H,f}$ and $R_{H, \eta }$, respectively, in Eqs. (11) and (12).

Note that the hydrodynamics is directly determined by the size/shape terms $f^*$ and $\nu$. Indeed, $a_T\equiv R_{H,f} = f^*/6\pi = 0.0488f^*$ and $a_I =(3/10\pi )^{1/3} (\nu V)^{1/3}=0.770 (\nu V)^{1/3}$. We emphasize, again, that the results of theoretical or computational calculations from hydrodynamic models just give $f^*$ and $\nu$, as well as $a_T$ and $a_I$, and the evaluation of observable hydrodynamic properties is performed subsequently.

The equivalent radii can be defined for several other hydrodynamic properties as well as those from other properties—particularly from scattering techniques. That is the case for the radius of gyration, $R_g$. Thus, another equivalent radius, $a_G$, is defined as the radius of a sphere having the same $R_g$ as the particle under consideration, and is formulated as

13 $$\begin{aligned} a_G \equiv \sqrt{5/3} R_g = 1.291 R_g \end{aligned}$$

$a_G$ is a bit—just about 30%—larger than $R_g$.

Another geometric property of the particle is its volume, and another equivalent radius is the volume-equivalent radius, $a_V$, i.e., the radius of a sphere having the same volume, *V*, as the particle. Since *V* is related to *M* through the specific volume, ${\bar{v}}$, as $V= {\bar{v}} M/N_A$, the definition is

14 $$\begin{aligned} a_V \equiv \biggl ( \frac{3 V}{4 \pi } \biggr )^{1/3}= \biggl ( \frac{3 M {\bar{v}}}{4 \pi N_A} \biggr )^{1/3} \end{aligned}$$

An important feature of any of the equivalent radii is that they depend essentially on the size and shape or conformation of the solute particles, being independent of the other solute/solvent properties (temperature, solvent viscosity $\eta _0$, and density $\rho$), provided that these properties do not modify the particle’s structure.

In particular, the hydrodynamic radii $R_{H,f}\equiv a_T$ and $R_{H,\eta } \equiv a_I$ are, as indicated above, the direct result from just the geometry of the model, and of course, of the modelling and computational procedures. Thus, if one wishes to compare the performance of alternative procedures, this fact its relevant: the comparison could be made in the most direct way, i.e., comparing the values of radii, which are their outcome.

Also, the analysis of experimental data, by comparison of experimental and calculated values, could be made transforming first the experimental *s*, *D*, and $[\eta ]$, with the use of experimental *T*, $\eta _0$, $\rho$, *M* and ${\bar{v}}$, into *experimental radii* $R_{H,f}$ and $R_{H,f}$. In the workflow of Fig. 1, this would be like inverting the direction of some arrows. Nothing is really wrong with the conventional protocol, but this one has some advantages, indicated next.

For instance, in the joint analysis of two or more properties, the experimental values of *s*, *D*, $[\eta ]$, etc, could be transformed into $R_{H,f}$ and $R_{H,\eta }$; both are in units of length. The analysis can include equivalent radii from other properties. This is the basis of the Hydfit global fitting scheme described in next section.

Another important feature is that the different $a_X$’s take values that are quite close to each other. Therefore, the *ratios of radii*, XY=$a_X/a_Y$, are all close to unity. It is also evident that any ratio can be formed by combining two others as XY=XZ/YZ.

In Eqs. (11) and (12), we use deliberately the notations $R_{H,f}$ and $R_{H,\eta }$, because *these two hydrodynamic radii are different*. This circumstance is generally ignored, thinking that they are the same value, simply denoted as $R_H$, such that experimental data of $R_{H,f}$ and $R_{H,\eta }$ would be analyzed in the same way, or values for one of them would be used to calculate the other property; for instance, $R_{H,f}$ would be used to calculate $[\eta ]$.

**Fig. 2:**
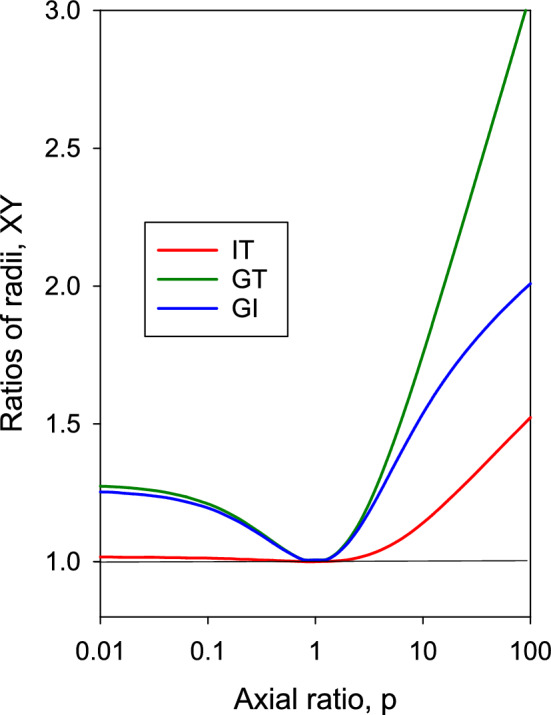
Ratios of radii IT, GT and GI, for revolution ellipsoids as functions of the axial ratio, $p=a/b$, calculated from the equations of Perrin (1934) and Simha (1940). Results from program EllypCylin (García de la Torre and Hernández Cifre 2020)

The fact that $R_{H,f}$ and $R_{H,\eta }$ are different is found in results of hydrodynamic calculations, and does not come from the possible deficiencies of the models or computation; instead, it can be demonstrated with the exact analytical expressions for ellipsoids. Figure 2 presents ratios of radii of ellipsoids GT$\equiv a_G/a_T$, GI$\equiv a_G/a_I$, and IT$\equiv a_I/a_T=$GI/GT, in a wide range of axial ratios, from very oblate to very prolate ones. The GT and GI ratios are the combinations of *f* and $[\eta ]$, respectively, with the radius of gyration, which is given for biaxial ellipsoids by $R_g^2=(a^2+2b^2/5)$, with $a_G=\sqrt{(a^2+2b^2)/3}$. Their values for ellipsoids, shown in Fig. 2, are obtained with program EllipCylin, presented in our previous work (García de la Torre and Hernández Cifre 2020).

As shown in Fig. 2, the ratio IT, given by

15 $$\begin{aligned} \text{ IT } \equiv \frac{a_I}{a_T} \equiv \frac{R_{H, \eta }}{R_{H, f}}= & \frac{6 \pi \eta _0 D}{k_B T} \biggl ( \frac{3 M [\eta ]}{10 \pi N_A} \biggr )^{1/3} \nonumber \\= & \frac{6 \pi \eta _0 s}{1-{\bar{v}} \rho } \biggl ( \frac{N_A}{M} \biggr )^{2/3} \biggl ( \frac{3 [\eta ]}{10 \pi } \biggr )^{1/3} \end{aligned}$$

while remaining practically equal to unity for oblate ellipsoids $(p<1)$, departs remarkably from unity for prolate ellipsoids $(p>1)$. For long prolate ellipsoids, $R_{H,\eta }$ is appreciably greater than $R_{H,f}$which confirms the inequality of the two hydrodynamic ratios. Nonetheless, in our experience, the IT ratio does not depart appreciably from unity except for very elongated rigid structures; except in such cases, for many particles, either rigid or flexible (as for flexible chains; see next section), IT is in the range 1.00–1.10.

This fact provides a useful procedure to estimate molecular weights from an estimate of IT and measurements of $[\eta ]$ and *f* obtained from *s* or from *D* (nowadays, there are simple, effective, bench-top sized instrumentation for viscometry and dynamic light scattering which can afford such determination). As a rule of thumb, we can take approximately IT $\approx$ 1.05. With this value in Eq. (15), we have (in CGS units):

16 $$\begin{aligned} M= & 2.86\times 10^{-27} \biggl ( \frac{T}{\eta _0 D} \biggr )^3 \frac{1}{[\eta ]} \nonumber \\= & 1.41\times 10^{25} \biggl ( \frac{\eta _0 s}{1-{\bar{v}} \rho } \biggr )^{3/2} [\eta ]^{1/2} \end{aligned}$$

and *M* can be evaluated with an uncertainty not exceeding 15%.

The applicability of Eq. (15) for the IT ratio, and its sequel equation (16) for an approximate estimation of M, is that of the basic equations, where they come from: the classical Einstein equations for *D* and $[\eta ]$: sufficiently dilute solutions of uncharged macromolecules, or polyelectrolytes with electrostatic interactions shielded at high ionic strength. Equation (16) bears some resemblance, and has similar applicability, to the famous Svedberg equation to obtain *M* from *s* and *D*, which is exact, while Eq. (16) is an approximation. Another noticeable difference is that Eq. (16) can be used combining $[\eta ]$ with *D*, without requiring ${\bar{v}}$, which may not be accurately known or even unavailable, as it could happen for particles with variable or unknown composition. The essential limitation of Eq. (16) is that its acceptable accuracy does not hold for particles showing a very long and elongated overall conformation, which would have an IT value beyond the above mentioned range, as evidenced by the values for very elongated ellipsoids (Fig. 2).

The ratios combining solution properties with the particle’s volume, XV$\equiv a_X/a_V$, are also important. As defined, the ratio TV$\equiv a_T/a_V$ turns out to be the same as the frictional ratio, $f/f_0$, and the ratio IV$\equiv a_T/a_V$ is equal to $2\nu /5$. Both ratios of radii are, as any others, equal to 1 for a spherical particle, and farther away from unity for any other case. Knowing these ratios for a model of the particle (for instance, a cylinder for a disk-like or rod-like particle (García de la Torre and Hernández Cifre 2020; Ortega and García de la Torre 2003)), $a_V$ is calculated from *M* and ${\bar{v}}$ using Eq. (14). Then, $a_T$=TV$\cdot a_V$, $a_I$=IV$\cdot a_V$, and the translational coefficients or the intrinsic viscosity can be obtained therefrom.

**Fig. 3:**
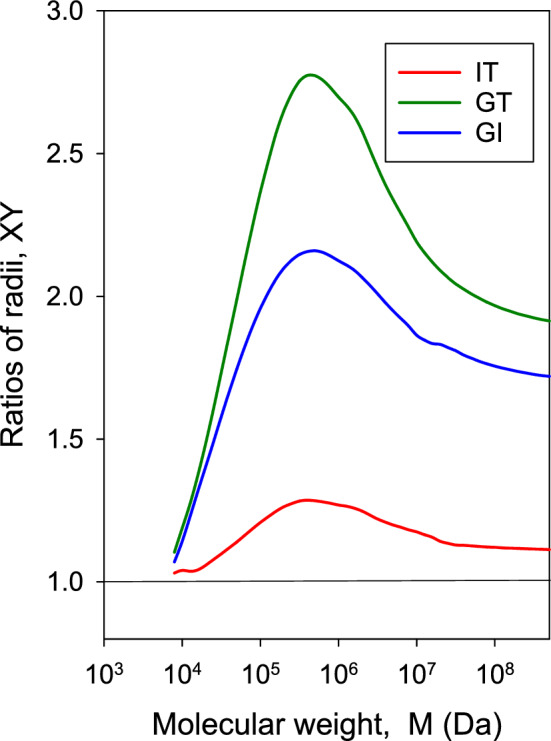
Ratios of radii IT, GT and GI for the wormlike chain model with the parameters corresponding to dsDNA ($P=56$ nm, $d = 2.3$ nm and $M_L$ Da/nm), for a wide range of molecular weights. Results from program Worm-2 (García de la Torre and Hernández Cifre 2020)

For linear macromolecular chains, the wormlike (WC), Kratky–Porod model is a most useful representation. The wormlike chain has contour length *L*, mass-per-unit length $M_L = M/L$, cross-sectional diameter *d*, and the degree of flexibility is gauged by the persistence length, *P*. This model covers the complete range of flexibility: when $L<<P$, the conformation is a rigid, straight rod, and when $L>>P$, it is a fully flexible random coil. A computational study of the WC model has been presented by our group (Amorós et al. 2011). At the core of this study was a MCRB calculation of hydrodynamic properties to obtain the ratios of radii GT and GI. $R_g$ was either calculated by the Benoit–Doty equation for ideal chains—without excluded volume (EV) effect—or by Monte Carlo simulation of conformations including EV. Using $R_g$ and these ratios in Eqs. (13), (11), and (12), the hydrodynamic properties could be calculated. Our programs Worm-1 and Worm-2 (García de la Torre and Hernández Cifre 2020) can be employed to calculate radii, ratios and measurable properties as $R_g$, *D*, *s*, $[\eta ]$, and others, for wormlike macromolecules with given values of *P*, *d*, and *L* or *M* and $M_L$ (Fig. 3).

### The Hydfit global fitting scheme: application to dsDNA

Yet another important application of the equivalent radii and their ratios is the determination of parameters of macromolecular models in a global fit of, not just a single property, but also, jointly, a set of various properties, such as $R_g$, *D*, *s*, $[\eta ]$, etc. Different properties have different dependencies on particle’s size. Thus, *s* or *D* are determined by a linear dimension (some hydrodynamic radius), low-angle scattering gives a square dimension (the square radius of gyration, $R_g^2$), and solution viscosity, expressed as an intrinsic viscosity, is related to some effective volume of the particle (Eq. (5)). Our global-fitting Hydfit scheme, implemented in programs Single-Hydfit and Multi-Hydfit, treats the various properties in a consistent way, using equivalent radii $a_X$ obtained from the experimental measurements of properties, and combined with the calculated values for the macromolecular model in a target function:

17 $$\begin{aligned} & \Delta ^2 (p_1, p_2,...) = \nonumber \\ & \quad \frac{1}{n_{species}} \sum _{species} \frac{1}{n_X} \sum _{X} \biggl (\frac{a_{X} - a_{X,ref}}{a_{X,ref}} \biggr )^2, \nonumber \\ & \quad \Delta _{\%} = 100\sqrt{\Delta ^2} \end{aligned}$$

$(p_1, p_2,...)$ are the parameters to be fitted by minimization of $\Delta$. The sum is extended over $n_{species}$ treated jointly. They could be a series of individual species (for instance, a collection of proteins) or fractions with varying molecular weight of the same polymer, etc. Then, there is a sum over the $n_X$ properties available for each component. The $a_X$’s are the equivalent radii calculated with the $(p_1, p_2,...)$ parameters, and the reference values will typically be the equivalent radii for experimental data, or maybe those computed with other model which is being compared. Note that the value of the percent root-mean-square deviation, $\Delta _{\%}$, is a useful indicator of the agreement between the two sets.

A paradigmatic example of the model parameter fitting is the Multy-Hydfit analysis of double-helical (double-stranded) DNA, dsDNA, in terms of the WC model by Amorós et al. (2011), with the parameters being the persistence length, *P*, the hydrodynamic diameter, *d*, and the mass-per-unit length, $M_L$. In their work, a compilation of experimental values of *s*, *D*, $[\eta ]$ and $R_g$ for dsDNA samples, from a dodecamer with $M \approx 8 \times 10^3$ Da to DNA of bacteriophage T2 with $M \approx 1.3\times 10^8$ Da, was carried out. This amounts to a number of base pairs, $n_{bp}$, from 12 to $2 \times 10^5$. The global analysis of such ample range included a large number of samples, with a total of 174 property vs. *M* data. The analysis yielded $P=56 \pm 2$ nm, $d = 2.3 \pm 0.1$ nm and $M_L= 1950 \pm 40$ Da/nm. The result for *P* is quite close to the persistence length of dsDNA of 50 nm that was being reported in studies with a more limited set of data.

Particularly remarkable is the agreement of *d* and $M_L$ with the structural, Watson–Crick values (1953). The diameter of the bare double helix is about 2.0 nm, and it could be approximately 0.3 nm wider if one accounts for a layer of hydration. In the Watson–Crick structure of the double helix, the rise per base pair is $l_{bp} = 0.34$ nm, so that the contour length of the wormlike DNA chain is $L = l_{bp} n_{bp}$. The molecular weight of a dsDNA with $n_{bp}$ base pairs is $M = M_{bp}n_{bp}$, where $M_{bp}$ is taken as the average molecular weight of a base pair. The value usually found in the literature is $M_{bp}=660$ Da (Stephenson 2003), although one sometimes finds $M_{bp}=619$ Da (Dolezel et al. 2003). The reason is that the former value refers to the bare DNA polyanion in salt, usually 0.1–0.5 M NaCl solution, while the latter is for the undissociated nucleic acid. Some amount of Na$^+$ bound to the double helix may explain the difference. We opt here for a compromise value, $M_{bp} \approx 640$ Da. Then the mass per unit length in the Watson–Crick double helix would be $M_L = 640/0.34 = 1882$ Da/nm. Our result, 1840 Da/nm is in very good agreement (with 660 Da/nm, our result would be exact). This important application, resulting in an excellent reproduction of the Watson–Crick parameters, shows how measurements of hydrodynamic and scattering properties of macromolecules in dilute solution, treated with modern modelling/computational methods, can be so powerful for structural determinations.

Further details of the Hydfit global fitting scheme and other examples can be found in previous papers (García de la Torre and Hernández Cifre 2020; Ortega and García de la Torre 2007; Ortega et al. 2011a).

### Other size/shape/conformation dependent combinations of properties

The idea of combining two or more hydrodynamic coefficients and other solution properties in quantities which would be indicators of the shape or conformational aspects was indeed formulated in the early years of macromolecular hydrodynamics. In the first 1950’s, Flory was working out a theory for solution properties of flexible, random-chain polymers (Flory and Fox 1951; Mandelkern and Flory 1952; Flory 1953), finding that the exponents of the property-*M* scaling laws for the friction coefficient, $f \propto M^{\alpha _f}$, and the radius of gyration, $R_g \propto M^{\alpha _g}$, had the same value, $\alpha _g = \alpha _f \equiv \alpha$ (the sign $\propto$, not to be confused with $\alpha$, means proportionality). Therefore, the ratio of these two quantities would be independent of *M*, and Flory formulated the numerical parameter

18 $$\begin{aligned} P_0= & \frac{f}{6^{1/2}\eta _0 R_g}=\frac{kT}{6^{1/2}\eta _0 R_gD} \nonumber \\= & \frac{M (1-{\bar{v}} \rho )}{6^{1/2} N_A \eta _0 R_g s} \end{aligned}$$

(The original notation is *P*, which is used in this paper for another purpose).

Also, the exponent in the power-law for the intrinsic viscosity of such chains was $[\eta ]\propto M^{\alpha _\eta }$, with an exponent $\alpha _\eta = 3 \alpha -1$. Therefore, $[\eta ]M \propto M^{3\alpha }$ and $[\eta ]M/R_g^3$ is independent of *M*, so Flory formulated another numerical parameter:

19 $$\begin{aligned} \Phi =\frac{[\eta ]M}{6^{3/2}R_g^3} \end{aligned}$$

According to the conformational theory of random, fully flexible chains, the common value $\alpha$ depends on the thermodynamic quality of the solvent. The Flory theory predicts that in the so-called Gaussian or ideal-chain regime, at the $\Theta$-temperature in poor solvent conditions, $\alpha =1/2$. When dealing with biomacromolecules in aqueous solution, the results for chains in good solvents with excluded-volume interactions would be more realistic; in such conditions $\alpha \approx 0.60$. More recently, the numerical values of the Flory parameters have been evaluated from MCRB simulations. For the ideal chain, $P_0=6.0$ and $\Phi =2.53 \times 10^{23}$ (García de la Torre et al. 1984; Freire et al. 1986), and for chains in good solvents with excluded-volume interactions, $P_0=5.3$ and $\Phi =1.9 \times 10^{23}$ (García Bernal et al. 1991). In our scheme, the Flory parameters are related to the ratios of radii as

20 $$\begin{aligned} P_0 = \frac{\sqrt{10} \pi }{GT} = \frac{9.93}{\text{ GT }} \end{aligned}$$

and

21 $$\begin{aligned} \Phi = 5^{1/2}2^{-1/2}3^{-4} \pi N_A / (GI)^3 = \frac{9.23 \times 10^{23}}{\text{(GI) }^3} \end{aligned}$$

In terms of ratios of radii, from Eqs. (20) and (21), GT=1.65 and GI=1.53 in an ideal solvent, and GT=1.87 and GI=1.69 in a good solvent. We note that these *universal* quantities are valid for any fully flexible polymer and constants for any sufficiently high-molecular-weight polymer.

The theoretical results for the scaling exponents and the formalism of Flory and coworkers indicated that a further compound quantity, $(M[\eta ])^{1/3}f^{-1}$, should be also independent of *M*, with the same value for any linear polymer that adheres to the random coil model. Furthermore, its values would be simply proportional to the solvent viscosity $\eta _0$. Then, they defined a parameter combining *M*, $[\eta ]$ and *f*, $\beta = \eta _0 (M[\eta ]')^{1/3}f^{-1}$.By that time, it was a common practice to express the intrinsic viscosity in units of dL/g (what we denote as $[\eta ]'= [\eta ]/100$), while the rest of quantities would be given usually in CGS units. To avoid confusions, in the modern literature the definition is

22 $$\begin{aligned} \beta \equiv \frac{M^{1/3}[\eta ]^{1/3} \eta _0}{100^{1/3} f} \end{aligned}$$

which would be another universal parameter for flexible, linear chain polymers. Indeed, $\beta$ is simply a further combination of the two other Flory parameters, $\beta \equiv 100^{-1/3} \phi ^{1/3} /P_0$. From the above indicated values of $P_0$ and $\Phi$, we have $\beta = 2.28 \times 10^{6}$ in $\Theta$-conditions, and $\beta = 2.34 \times 10^{6}$ in good solvents. As the two others, the $\beta$ parameter is immediately related to the IT ratio,

23 $$\begin{aligned} \beta = \biggl ( \frac{\pi N_A}{30} \biggr )^{1/3} \frac{\text{ IT }}{6 \pi } = 2.112 \times 10^6 \cdot \text{ IT } \end{aligned}$$

The quantities $P_0$ and $\Phi$ are not only applicable to flexible macromolecules. Equations (18) and (19) can be considered as just definitions by which values (either experimental or calculated) for any particle can be combined. For instance, in the case of a rigid, compact, spherical particle, it is easily found that $P_0=9.93$ and $\Phi =9.23 \times 10^{23}$, and their combination yields $\beta =2.112 \times 10^6$ with obviously GT=GI=IT=1, as every XY=1 for a sphere.

Inspired for the work done within Flory’s group at Cornell University, H. Scheraga had the idea of applying the definition of $\beta$, previously worked out by Mandelkern and Flory, to models for proteins, so that $\beta$ is well-known as the Scheraga–Mandelkern parameter (Scheraga and Mandelkern 1953). About his work in the early 1950’s, Scheraga wrote (1992): *I joined... Cornell University in 1947... I was disappointed to learn that Jack Kirkwood, a foremost statistical mechanician, had left... However, this disappointment vanished when Paul Flory (the world’s leading polymer physical chemist) came to Cornell... At Cornell, I began to think about hydrodynamic properties of polymers. Inspired by association with Flory, Leo Mandelkern and I proposed a procedure to deduce the dimensions of protein molecules from their hydrodynamic properties, using rigid ellipsoids of revolutions as models to describe these otherwise intractable entities. *

More details about the use of the Scheraga–Mandelkern parameter for rigid particles are described at the end of this section.

While the physical chemistry of macromolecules in solution flourished around Cornell University in the US, activity in that field was also going on around St. Petersburg, in the USSR, lead by Tsvetkov (1953) is cited in the literature as an early report on this work, and a wide-reaching publication was (1984). These works proposed a certainly correct, but formally very different scheme for the analysis of properties using size/shape-dependent functions and parameters. Thus, the scheme begins introducing an *intrinsic diffusion* coefficient, [*D*], defined in Eq. (24):

24 $$\begin{aligned} & {[}D] \equiv \frac{D \eta _0}{ T} \nonumber \\ & {[}D] =\frac{k_B}{6 \pi } \frac{1}{R_{H,f}} =\frac{7.32 \times 10^{-18}}{a_T} \end{aligned}$$

The CGS unit of [*D*] is cm$^{-1}$. It is immediately clear that [*D*] is equivalent to the reciprocal of the hydrodynamic radius—$R_{H,f}$ also denoted as $a_T$—except for a numerical factor. Nowadays, particle-sizing instruments based on determinations of the diffusion coefficient—usually from DLS—and also (as emphasized previously) some recent computational tools, present results in the form of a hydrodynamic radius. Therefore, whatever the role of [*D*] is in a formalism for analysis of solution properties, we think that the formalism that employs $R_{H,f}$ or $a_T$ will be more familiar to users.

Similarly, these workers used in their formalism, for theoretical expressions and presentation of results, an *intrinsic sedimentation* coefficient, [*s*], defined as

25 $$\begin{aligned} & {[}s] \equiv \frac{s \eta _0}{1-{\bar{v}} \rho } \nonumber \\ & {[}s] =\frac{1}{6 \pi N_A} \frac{M}{R_{H,f}} = 8.81 \times 10^{-26} \frac{M}{a_T} \end{aligned}$$

The CGS unit of [*s*] is Da/cm. It is clear, again, that this other *intrinsic* coefficient is just the ratio of molecular weight to the hydrodynamic radius, multiplied by a numerical factor. In addition, this intrinsic coefficient can be written as $[s]=[D]M/R$, where *R* is the ideal gas constant, so that [*s*] is just another combination of the other intrinsic coefficient, [*D*], with *M*.

In 1984, Tsvetkov et al. (1984) proposed the use of what they called the *hydrodynamic invariant*. These authors were aware—with reference to the Mandelkern–Flory paper (1952)—of the fact that, as described above, the exponents of the property-*M* scaling were such that the quantity

26 $$\begin{aligned} & A_0 \equiv k_B \eta _0 (M[\eta ]')^{1/3}/f \equiv \nonumber \\ & 100^{-1/3} k_B \eta _0 (M[\eta ] )^{1/3}/f \end{aligned}$$

would be a universal quantity for linear, flexible random-coil polymer chains. These authors kept the early convention about using $[\eta ]'$ in dL/g; the right-hand side of Eq. (26) is written in CGS units for coherent notation.

In the Tsvetkov scheme, $A_0$ is a further combination of the intrinsic coefficients

27 $$\begin{aligned} A_0= & 100^{-1/3}(M[\eta ])^{1/3} [D] \nonumber \\ = & 100^{-1/3} R [s] [\eta ]^{1/3} M^{-2/3} \end{aligned}$$

By comparing Eq. (26) with Eq. (22), we see that the Tsvetkov invariant is just the $\beta$ Scheraga–Mandelkern parameter multiplied by the Boltzmann constant, $A_0=k_B \beta$.

The use of the Tsevetkov formalism in the analysis of experimental data for synthetic and biological polymers in solution is illustrated in many publications [see, for instance papers by Pavlov et al. (1990, 2010, 2016), Tsevetkov et al. (2017, 2017), Grube et al. (2020b; a), etc]. The equivalence between the Tsevetkov intrinsic coefficients, [*s*] and [*D*], and the Flory parameters, *P* and $\Phi$, was presented years ago by Pavlov et al. (2009).

In the field of rigid-particle hydrodynamics, shape-dependent compound quantities are very useful for the interpretation of experimental results. As mentioned above, the classical parameters $\beta$, $\phi$ and $P_0$ can be formulated for any rigid particle. A remarkable contribution in this field is the work of Harding and his coworkers, who extended the hydrodynamic theory to cover many other solution properties of ellipsoids (Harding and Rowe 1982)—treating even triaxial ellipsoids (Harding et al. 1981)—and formulated a number of shape-dependent, universal functions of the axial ratio, *p*, (Harding 1980a, b, 1989; Harding et al. 1992; Harding 1995). Thus, they published the ELLIPS package of programs (Harding et al. 1997) to manage the use of ellipsoidal models in the analysis of experimental properties (Harding and Cölfen 1995). The program SOLPRO (Carrasco et al. 1998) has an interface with HYDRO to compute the Harding’s shape functions from results obtained with bead modelling. Indeed, the packages ELLIPS and HYDRO are complementary tools and have been used jointly (Carrasco et al. 1999; García de la Torre and Harding 2013). Our new program EllipCylin (García de la Torre and Hernández Cifre 2020), which computes the equivalent radii and ratios of radii for both ellipsoids and cylinders, was constructed with a firm basis on Harding’s developments.

## Hydrodynamics in analytical ultracentrifugation

### Elementary aspects

After the above commented fundamental studies of Einstein and Perrin, the first of the two landmarks that definitive established the theoretical and experimental basis of macromolecular hydrodynamics was the invention of the analytical ultracentrifuge by Svedberg (1923, 1940). The other one, which is not treated here but deserves to be cited, was the development by Staudinger (1933) of concepts and methods related to the dilute-solution viscosity of compounds that were confirmed to be *macro-molecules*.

The most basic theory of analytical ultracentrifugation (AUC) lies on the balance between the two kinds of forces, centrifugal and frictional, experienced by the solute particles. As described in elementary textbooks and monographs (Van Holde et al. 1998; Atkins and de Paula 2010; Serdyuk et al. 2007; Hiemenz and Lodge 2007; Schuck et al. 2016), this balance is determined by:

28 $$\begin{aligned} m^{(b)}g' -fv = 0 \end{aligned}$$

where $m^{(b)}=m(1-{\bar{v}} \rho )$ is the buoyant mass of a particle with mass $m=M/N_A$ and specific volume ${\bar{v}}$ in a fluid of density $\rho$ (nearly equal to the solvent density in a dilute solution). In Eq. (28), $g'=\omega ^2 r$ is the centrifugal acceleration (analogous to the gravitational acceleration, *g*) of the particle when it is at distance *r* from the rotor, gyrating with angular velocity $\omega$. Its instantaneous translational velocity is $v=d r/d t$, i.e., the time-derivative of the radial position. Then, the equation of motion, Eq. (28), can be rewritten as

29 $$\begin{aligned} \frac{d r}{d t} = \omega ^2 \frac{m^{(b)}}{f} r \end{aligned}$$

which is a simple differential equation whose solution is

30 $$\begin{aligned} r(t) = r(t_0) \exp \biggl [ \omega ^2 \frac{m^{(b)}}{f} (t-t_0) \biggr ] \end{aligned}$$

where $r(t_0)$ is the position of the particle at any initial time $t_0$, and *r*(*t*) is the position at any later time, *t*. This equation can be easily written as

31 $$\begin{aligned} \ln \frac{r(t)}{r(t_0)}= \omega ^2 s (t-t_0) \end{aligned}$$

where the quantity *s*, which is proper of properties of the solute and solvent, defined as

32 $$\begin{aligned} s \equiv \frac{m^{(b)}}{f} = \frac{1}{6\pi }\frac{m^{(b)}}{\eta _0 R_{H,f}} \end{aligned}$$

is universally named the sedimentation coefficient. When the buoyant mass is written in the terms on which it depends, then the definition that is customarily presented is Eq. (7). However, it is wise to keep in mind the concept implicit in Eq. (32): the sedimentation coefficient is just the ratio of the buoyant mass to the friction coefficient. Furthermore, the quantity

33 $$\begin{aligned} s\eta _0 = \frac{1}{6\pi }\frac{m^{(b)}}{R_{H,f}} = 0.053 \frac{m^{(b)}}{R_{H,f}} \end{aligned}$$

is just the ratio of the buoyant mass to the hydrodynamic radius coming from the Stokes friction equation.

Based on this formalism, the analysis of velocity ultracentrifugation data can be made in a straightforward manner, taking into account that the particles that are at the boundary at time $t_0$, will be all at the boundary at time *t*. At $t=0$ the boundary is at the meniscus, placed at $r_m$. Then the position of the boundary at time *t* will be determined by

34 $$\begin{aligned} \ln \frac{r(t)}{r_m}= \omega ^2 s t \end{aligned}$$

Measuring the position of the boundary, *r*(*t*), at some times during the centrifugation run and plotting $\ln [r(t)/r_m]$ vs. time, t, the sedimentation coefficient is easily determined from the slope = $\omega ^2 s$.

### Inclusion of the effect of diffusion. The Lamm equation and Brownian dynamics simulation

The previous description is incomplete, as it does not consider, along with centrifugal and frictional contributions, another essential contribution to particle motion, which is the diffusion caused by the concentration gradients originated by the centrifugation. Indeed, it is valid when those two first contributions are sufficiently large, because either $m^{(b)}\omega ^2$ is sufficiently large and/or *f* is sufficiently small (i.e., when $\omega ^2s$ is sufficiently large). The effect of diffusion is a broadening of the sedimenting boundary. If it is just slightly broad, an approximate value of *s* can be determined from Eq. (34) following the time-course of a specific point within the boundary, e.g., the midpoint between the solvent and solution plateau.

The consideration of diffusion in the theory of sedimentation was made by inclusion of a term for a diffusive flux based on the Fick’s laws that describe macroscopic diffusion. This leads to the famous Lamm equation (1929), which can be written as

35 $$\begin{aligned} \frac{\partial c}{\partial t} = D \biggl [ \frac{\partial ^2 c}{\partial r^2} + \frac{1}{r}\frac{\partial c}{\partial r}\biggr ] - s \omega ^2 \biggl [ r \frac{\partial c}{\partial r} + 2c \biggr ] \end{aligned}$$

Note that Eq. (35) adds another molecular parameter to the treatment of sedimentation: the diffusion coefficient. In addition, the complexity of solving the differential equation is considerable, so that during the last years of the twentieth century there was intense activity to develop approximate methods to account for diffusion, which would allow the treatment of the wealth of data provided by modern AUC instrumentation. It was during the first years of the current twenty-first century when advanced computational hardware allowed the development of new software which would include an accurate numerical solution of the Lamm equation [see Ref. (Schuck 1998, 2000; Cao and Demeler 2005, 2008; Schuck 2016; Stafford and Sherwood 2004; Philo 2006; Chaturvedi and Schuck 2024) and references therein] that had been devised some years ago (Claverie et al. 1975). Programs SEDFIT (Schuck 2000, 2016) and ULTRASCAN (Demeler and Gorbet 2016) were available early in the 2000’s.

Apart from the implementation of the solution to the Lamm equations, these approaches faced the problem of providing the second molecular parameter, *D*, which, along with *s*, enters in these schemes. The initial solution was resorting to the classical frictional ratio *f*/*f*0 (the same as the Perrin function or the TV ratio o radii, *vide supra*) for which some previous knowledge or assumptions could provide estimations from the known or expected shape or conformation of the molecules. Having a value of *s*, the value of *D* required in the Lamm equation would be obtained from the relationship [Equation 3 in (Brown and Schuck 2006) with cite to Ref. (Schuck 2000)]:

36 $$\begin{aligned} D = \frac{\sqrt{2}}{18\pi } k_BT s^{-1/2} [\eta _0 (f/f_0)]^{-3/2} \biggl ( \frac{1-{\bar{v}} \rho }{{\bar{v}}} \biggr ) ^{1/2} \end{aligned}$$

or [Equation 3 in Ref. Brookes et al. (2010a)]:

37 $$\begin{aligned} D = RT \biggl [ N_A 18 \pi [\eta _0(f/f_0)]^{3/2} \biggl ( \frac{s {\bar{v}}}{2(1-{\bar{v}} \rho )} \biggr )^{1/2} \biggr ]^{-1} \end{aligned}$$

Another relationship is [Equation 7 in Ref. Beckdemir and Stellaci (2016), citing Brown and Schuck (2008)]:

38 $$\begin{aligned} f/f_0 = \biggl (\frac{\sqrt{2}}{18\pi }\biggr )^{2/3} \biggl ( \frac{k_BT}{D} \biggr )^{2/3} \frac{s^{-1/3}}{\eta _0} \biggl ( \frac{1-{\bar{v}} \rho }{{\bar{v}}} \biggr ) ^{1/2} \end{aligned}$$

In 2011, our group put forward a novel approach to predict sedimentation concentration profiles in non-interacting multicomponent samples. Instead of the macroscopic treatment of diffusion introduced in the Lamm equation with a term based on Ficks’s law, our approach was based on the microscopic Einstein’s description of diffusion as a manifestation of the Brownian motion. In our scheme, the motion of the solute molecules is the superposition of two terms, one of which is deterministic, caused by the centrifugal field, such that in a time interval, $\Delta t$, the radial displacement would be, according to Eqs. (11), (28), (31) and (32):

39 $$\begin{aligned} \Delta r_{sed}= & r(t) \biggr [ 1- \exp (s \omega ^2 \Delta t )\biggr ] \nonumber \\ = & r(t) \biggr [ 1- \exp \biggl ( \frac{m^{(b)}\omega ^2}{6 \pi \eta _0 R_{H,f}} \Delta t ) \biggr ) \biggr ] \end{aligned}$$

The second displacement is stochastic, based on the mean squared displacement of a Brownian particle in one direction (the radial one) during $\Delta t$:

40 $$\begin{aligned} \langle (\Delta r_{brow})^2 \rangle =2 D \Delta t = \frac{k_BT}{3 \pi \eta _0 R_{H,f}} \Delta t \end{aligned}$$

so that the final position after the time step would be

41 $$\begin{aligned} r(t+\Delta t) = r(t) + \Delta r_{sed} + \Delta r_{brow} \end{aligned}$$

Equations (39)–(41) are the basis of an algorithm for simulation of the trajectory of Brownian particles in a centrifugal field.

In the implementation of our BD algorithm, the input data of the solute particles are just $m^{(b)}$ and $R_{H,f}$. The working value of *s* is calculated from its definition, Eq. (32), with *f* and *D* given by the Stokes-Einstein equation. The only required, additional data are the rotor speed $\omega$, the temperature *T* and the solvent viscosity $\eta _0$ at that temperature. More details are described in our previous papers on this scheme (Díez et al. 2011; García de la Torre et al. 2018a). Our BD approach has been now adopted by other authors (Walter et al. 2015).

### The inverse problem: choosing variables in 2D data analysis

The Lamm equation solvers, or the Brownian dynamics simulation (Schuck 2000; Díez et al. 2011), are then available for calculating, from a given set of physico-chemical properties of solute and solvent and instrumental parameters, the signal profiles *z*(*r*, *t*) for an heterogeneous sample (*z* is an observable quantity linearly dependent on concentrations).

Then, such solutions are to be implemented in methods for the inverse problem, i.e., to analyze the experimental *z*(*r*, *t*) for the obtention of the desired molecular parameters of the present species. In the first developments, the primary result was a distribution of sedimentation coefficients, *c*(*s*), with information provided or obtained about their frictional ratios $f/f_0$ (Schuck 2000; Demeler 2005). Advanced numerical procedures, mainly non-linear least squares (NLLS) (Lawson and Hanson 1974), allowed the extension to the so-called 2D analysis, which provides a two-variables $c(s,f/f_0)$ distribution function (Brown and Schuck 2006; Brookes et al. 2010a). From the spots in a 2D plot, numerical results are extracted for the two properties, *s* and $f/f_0$, of each component in the sample.

In our opinion, in the schemes for analyzing AUC sedimentation experiments, the physical, numerical, computational aspects are pretty well-developed, but an aspect that may be considered is the choice of the structural and hydrodynamic parameters. About $f/f_0$, we have discussed above how it is a complex quantity whose handling or interpretation relies on a number of quantities: mass and specific volume, particle’s volume and hydrodynamic size and shape, or even hydration in frequent cases.

Even the sedimentation coefficient, *s*, is also a compound quantity that somehow depends on those various quantities. Certainly, *s* is a most significant property, extremely relevant in various senses. Its dependency on particle’s shape and composition makes it an excellent indicator of the identity of a macromolecular compound, and since the early years of molecular biology, the S (svedberg units) value has been profusely employed to nominate subunits, macromolecular complexes, etc (e.g., the 70 S ribosome, its small 30 S and large 50 S subunits, the 5 S, 16 S, 25 S RNAs, and so on). In addition, the value of *s* has a variety of practical applications—for instance, to estimate, in preparative ultracentrifugation, how long one has to centrifuge a sample to reach total sedimentation at the bottom of the cell. However, for the purpose of providing data about structure (shape, size, conformation, molecular properties, etc), the fact that *s* is a combination of geometry, mass, and densities of solute and solvent, poses some inconveniences in the interpretation of data.

To interpret the species corresponding to a spot with the $(s,f/f_0)$ coordinates in the 2D plot, the values ${\bar{v}}$ and $\rho$ should be known. And to transform the $c(s,f/f_0)$ distribution into a *c*(*s*, *D*) distribution, ${\bar{v}}$ should have the same value for all the components. This places some limitations in the analysis in terms of $c(s,f/f_0)$. There are cases when, even if ${\bar{v}}$ is the same, its value may be unknown *a priori*, and there are situations in multicomponent samples when ${\bar{v}}$’s are different and unknown for all the components. Such may be the case with hybrid, heterogeneous core–shell nanoparticles; a clear example is the data analysis from $c(s,f/f_0)$ distribution for DNA–gold (DNA–Au) hybrid particles (Urban et al. 2016), in which there were many components whose composition (DNA–Au stoichiometry) was variable and unknown. Some appropriate approaches for this situation for core–shell and drug-loaded nanoparticles (Demeler et al. 2014; Henrickson et al. 2021) have been successfully applied.

We have made it clear that the time course of sedimentation is determined, and, therefore, can be predicted and analyzed in terms of a reduced set of quantities: the hydrodynamic radius $R_{H,f}$, and the buoyant molecular weight $M^{(b)}=N_Am^{(b)}$, along with the solvent viscosity, $\eta _0$. Then, the analysis of sedimentation results can be made in terms of a $c(R_{H,f}, M^{(b)})$ distribution, thus identifying the $R_{H,f}$ and $M^{(b)}$ values of each component. *The hydrodynamic radius is, in this way, a primary result of the AUC experiment*, and since it is also the primary result of hydrodynamic-model calculation, it can be employed immediately for structural analysis.

Of course the friction, diffusion and sedimentation coefficients can be evaluated, for each component, without additional data, as $f=6\pi \eta _0 R_{H,f}$, $D=k_BT/f$, and $s=M^{(b)}/(N_Af)$, and reported. With values of ${\bar{v}}$ and $\rho$, the molecular weight will be obtained as $M=M^{(b)}/(1-{\bar{v}} \rho )$.

## Conclusions

Being well over 100 years, the field of macromolecular characterization based on dilute-solution properties is now living another “golden age”, brought by continuing advances in various aspects.

Thus, the classical theories of the physical chemistry of macromolecules in solution have covered now relevant problems, like those of hydrodynamic interactions or excluded volume. Such problems have been attacked still from the classical views, but with the possibility of finding their solutions with the help of computers of continuously increasing power. Certainly this has been a labour for theoreticians who would devise the way of treating the complexity of those problems, often by means of adequate models, and writing computer programs which implemented their solutions.

Nowadays, there is a number of methods for the calculation of solution properties of proteins and nucleic acids from their atomic-level or residue-level rigid models. The various methods may differ in the way of modelling the structure, the variants of hydrodynamic theory that they employ, details on numerical computation, etc. However, the general trend is that they all present a similar good performance in the predictions when compared to experimental data. The deviations, which must be evaluated in terms of the differences between the calculated equivalent radii ($a_T$, $a_I$, $a_G$,etc) and those obtained from the measured properties, are always of a few percent, within the expected uncertainty of their values obtained from experimental data (García de la Torre 2001b; García de la Torre and Hernández Cifre 2020). The good quality of the results predicted from quite different procedures is reassuring, as it confirms the reliability of present hydrodynamic theories and methods.

Experimental techniques are now also in pretty good conditions. For instance, simple, but efficient, benchtop-sized an moderately priced instrumentation is now available for measuring diffusion coefficients, *D*, and intrinsic viscosity, $[\eta ]$. The modern methodology of equivalent radii and ratios of radii can be applied to such joint measurements. The primary measurements of *D* and $[\eta ]$ can be transformed into values of hydrodynamic radii, $a_T$ and $a_I$. Then, this two values should be compared, keeping in mind that they may be different but not too much; their ratio $IT=a_T/a_I$ is, in many instances close to unity, as we have shown. In addition, an estimate of the molecular weight can be made easily from them, using Eq. (16).

In the particular field of analytical centrifugation, in addition to instrumental advances, an essential role is that of the methods for data analysis, which allow the extraction of a wealth of results from AUC experiments. As noted in the previous section, the complex core components of the programs for sedimentation analysis are well-developed, but some other pieces can be changed. Considering the sedimentation coefficient and the frictional ratio as the main quantities for carrying out the analysis and presentation of results could be somehow a reminiscence of traditional, previous procedures in the AUC field. We have proposed in this essay another choice, in which the hydrodynamic equivalent (Stokes) radius is, along with the buoyant mass, the quantities that are primary obtained.

## Data Availability

The paper is a review about existing theory and methodology. There is not new data.

## Appendix: A brief primer on particle hydrodynamics

This essay is intended to transmit some basic information and, mostly, essential concepts about macromolecular hydrodynamics. The authors assume that all its contents would be accesible to a wide readership, and particularly to practical users of the hydrodynamic experimentation and theory. Accordingly, some advanced physical topics and mathematical formalisms have been avoided. Nonetheless, the field is still certainly under active development. During the editorial review, it became apparent that some further detail in such aspects would be welcome by some readers, and such is the purpose of this Appendix.

### Hydrodynamics of an arbitrarily shaped particle. Intrinsic viscosity

The classical studies of Einstein included the viscosity of solutions. His famous result relates the viscosity increment, expressed as a linear dependence of solution-to-solvent viscosities ratio, i.e., the relative viscosity, $\eta _r \equiv \eta /\eta _0$, on the volume fraction of the solute, $\phi$:

42 $$\begin{aligned} \eta _r \equiv \eta / \eta _0 = 1 + \nu \phi \end{aligned}$$

where $\nu$ a is numerical factor, which takes the well-known value $\nu _{sph}=5/2$ for a spherical particle. The volume fraction is the ratio of volume-of-particles to volume-of-solution, and can be written as

43 $$\begin{aligned} \phi = \frac{\text{ mass-of-particles }}{\text{ volume-of-solution }} \frac{\text{ volume-of-particles }}{\text{ mass-of-particles }} \end{aligned}$$

Clearly, the first ratio in Eq. (43) is the mass concentration of the solute, and the second one is its specific volume, ${\bar{v}}$. Thus, $\phi = c {\bar{v}}$.

On the other hand, what is nowadays called intrinsic viscosity, $[\eta ]$, is the ratio of the specific viscosity, $\eta _{sp} \equiv (\eta - \eta _0)/\eta _0=\eta _r-1$, to concentration, *c*. Then, it follows that

44 $$\begin{aligned} {[}\eta ] = \nu {\bar{v}} \end{aligned}$$

For a particle of volume $V_p$ and mass $m=M/N_A$, the specific volume is ${\bar{v}}= N_A V_p/M$. Introducing now $\nu _{sph}$, it turns out that Eq. (44) can be rewritten as:

45 $$\begin{aligned} {[}\eta ] = \frac{N_A \nu _{sph}}{M} \frac{\nu }{\nu _{sph}} V_p = \frac{5}{2} \frac{N_A}{M} \nu V_H \end{aligned}$$

where

46 $$\begin{aligned} V_H = \frac{\nu }{\nu _{sph}} V_p = \frac{2}{5} \nu V_p \end{aligned}$$

is an effective, hydrodynamic volume of the particle, determined by the volume of the particle itself and its shape, which determines the Einstein coefficient $\nu$. Obviously, for a spherical particle, the effective volume is the volume of the particle itself. Thus, *the intrinsic viscosity is determined by the molecular weight and this size/shape-dependent hydrodynamic volume*. If one defines an equivalent radius for the intrinsic viscosity, $R_{H,\eta }$, as the radius of a sphere having a volume $V_H$, that is

47 $$\begin{aligned} R_{H,\eta } = (3V_H/4\pi )^{1/3} = \biggl ( \frac{3 M [\eta ]}{10 \pi N_A} \biggr )^{1/3} \end{aligned}$$

then Eq. (12) in Sect. 6 is obtained.

### Biophysics at low Reynolds number

Any particle moving in a fluid (the solvent, in a solution or dispersion) experiences some resistance, which can be of one of the two kinds that can be well-distinguished. In one of them, the laminar regime, the counterflow of the fluid caused by the particle motion presents well-defined layers which do not mix with their neighbors. In the other one, the turbulent regime, the motion of the fluid shows eddies and other irregular features, thus presenting a somehow chaotic aspect. The regime that is presented is determined by a dimensionless quantity named Reynolds number (although it was introduced by Sir Georges Stokes) and depends on the (dynamic) viscosity of the solvent, $\eta _0$, the velocity of the particle, *v*, its density, $\rho _p$, and its geometry expressed by a characteristic size, *L*. The particle Reynolds number is

48 $$\begin{aligned} \text{ Re}_p = \frac{v L \rho _p}{\eta _0} \end{aligned}$$

Fluid mechanics theory tell us that in low-Reynolds-number conditions, when $\text{ Re}_p < 1$, the hydrodynamic behavior of the particles is that corresponding to the laminar regime. Among macromolecules, macromolecular complexes and nanoparticles, the typical sizes are up most, say, $5 \times 10^{-5}$ cm. With a particle density of, say, $\rho _p \approx$ 2 g/cm$^3$, (${\bar{v}} \approx 0.5$ cm$^3$/g, like that of nucleic acids) in an aqueous solvent with $\eta _0 \approx 0.01$ poise, for a particle moving with velocity *v*, we have Re $\approx v$(cm/s)/100. In a sedimentation experiment, a very large particle having, say, a sedimentation coefficient $s=5000$ S, placed at 7 cm of the rotor axis, and with a rotor speed of 50 000 rpm, the velocity is $v \approx 0.09$ (cm/s) and $\text{ Re } \approx 9 \times 10^{-4}$.

Let’s just mention that hydrodynamics at low Reynolds number is important in other fields of biology; particularly it governs the swimming of flagellated micro-organisms (such as spermatozoa and some bacteria) as described in the beautiful review by Purcell (1977). Bead models have also been used to relate the swimming velocity to the structure and dynamics of these biological particles [see Fig. 1 in Ref. García de la Torre and Bloomfield (1977b)].

### Hydrodynamics of an arbitrarily shaped particle. Translational diffusion

Stokes demonstrated that the friction coefficient, *f*, which is the ratio of the frictional force experienced by the particle to its velocity, in low-Reynolds-number condition, is always proportional to the solvent viscosity, $\eta _0$, as expressed in Eq. (1). The proportionality constant, i.e., the reduced friction coefficient, $f^*$, depends only on the geometry of the particle, and is equal to $6 \pi R$ for a sphere of radius *R*.

For a particle of arbitrary shape, which would be translating or/and rotating, the generalized velocity is expressed by a vector of six components: three for the linear velocity, ${{\textbf{U}}}$, and three for the angular velocity, ${{\textbf{O}}}$. Similarly, the generalized force vector has six components: three for the force, ${{\textbf{F}}}$, and three for the torque, ${{\textbf{T}}}$. In low-Reynolds-number regime, this vector is linearly related to the generalized velocity by means of a $6\times 6$ generalized frictional-resistance matrix (Brenner 1967; Happel and Brenner 1973),

49 $$\begin{aligned} \begin{pmatrix} {{\textbf{F}}} \\ {{\textbf{T}}} \end{pmatrix} = \begin{pmatrix} {\mathbf \Xi }^{tt}& {\mathbf \Xi }^{tr,T} \\ {\mathbf \Xi }^{tr}& {\mathbf \Xi }^{rr} \end{pmatrix} {\mathbf \cdot } \begin{pmatrix} {{\textbf{U}}} \\ {{\textbf{O}}} \end{pmatrix} \end{aligned}$$

which is partitioned in four $3\times 3$ blocks: ${\mathbf \Xi }^{tt}$ and ${\mathbf \Xi }^{rr}$ associated with translation and rotation, respectively, and ${\mathbf \Xi }^{tr}$ and its transpose ${\mathbf \Xi }^{tr,T}$, which arise from translation-rotation coupling. In a more compact notation, Eq. (49) can be written as

50 $$\begin{aligned} {{\textbf{F}}}_{(6)} = {\mathbf \Xi }_{(6\times 6)} {\mathbf \cdot } {{\textbf{U}}}_{(6)} \end{aligned}$$

A reciprocal relationship giving velocities from forces and torques can be written as:

51 $$\begin{aligned} {{\textbf{U}}}_{(6)} = {{\textbf{M}}}_{(6\times 6)} {\mathbf \cdot } {{\textbf{F}}}_{(6)} \end{aligned}$$

where the mobility matrix, ${{\textbf{M}}}_{(6\times 6)}$, is the inverse of the resistance matrix,

52 $$\begin{aligned} & {{\textbf{M}}}_{(6\times 6)} \equiv \begin{pmatrix} {{\textbf{M}}}^{tt}& {{\textbf{M}}}^{tr,T} \\ {{\textbf{M}}}^{tr}& {{\textbf{M}}}^{rr} \end{pmatrix} = {\mathbf \Xi }_{(6\times 6)}^{-1} \nonumber \\ & \quad = \begin{pmatrix} {\mathbf \Xi }^{tt}& {\mathbf \Xi }^{tr,T} \\ {\mathbf \Xi }^{tr}& {\mathbf \Xi }^{rr} \end{pmatrix}^{-1} \end{aligned}$$

Furthermore, an essential result in fluid mechanics is that, in low-Reynolds-number conditions, all the $6\times 6$ components of ${\mathbf \Xi }_{(6\times 6)}$ must be simply proportional to the solvent viscosity, $\eta _0$ (such as the Stokes frictional coefficient). Then, the solvent viscosity can be factored out of ${{\textbf{M}}}_{(6\times 6)}$, defining a reduced mobility matrix

53 $$\begin{aligned} {{\textbf{M}}}_{(6\times 6)}^{*} = \eta _0 {\mathbf \cdot } {{\textbf{M}}}_{(6\times 6)} \end{aligned}$$

At this point, we emphasize that the reduced mobility ${{\textbf{M}}}_{(6\times 6)}^{*}$ depends only on the geometry (shape and dimensions) of the particle, i.e., it can obtained with theoretical or modelling calculations.

The hydrodynamic theory of macromolecules and nanoparticles is a combination of the classical fluid mechanics with the statistical concepts developed by Einstein to explain observable hydrodynamic properties in terms of microscopic Brownian motion. The scalar Einstein equation $D=k_B/f$ is transformed into a generalized Einstein relationship, that gives a $6 \times 6$ diffusion matrix, ${{\textbf{D}}}_{(6\times 6)}$ (Brenner 1967):

54 $$\begin{aligned} {{\textbf{D}}}_{(6\times 6)} \equiv \begin{pmatrix} {{\textbf{D}}}^{tt}& {{\textbf{D}}}^{tr,T} \\ {{\textbf{D}}}^{tr}& {{\textbf{D}}}^{rr} \end{pmatrix} = k_B T {\mathbf \cdot } {\mathbf \Xi ^{-1}_{(6\times 6)}} \end{aligned}$$

and, in terms of the mobility matrix,

55 $$\begin{aligned} {{\textbf{D}}}_{(6\times 6)} = k_B T {\mathbf \cdot } {{\textbf{M}}_{(6\times 6)}} = (k_B T/\eta _0) {\mathbf \cdot } {{\textbf{M}}^*_{(6\times 6)}} \end{aligned}$$

During translational Brownian motion, governed by block ${{\textbf{D}}}^{tt}$, the particles experience a Brownian tumbling which is very fast when compared to the time scales of spontaneous macroscopic diffusion and sedimentation in AUC experiments, and therefore, the observable, effective diffusion coefficient corresponds to an average over random orientations, which is given by

56 $$\begin{aligned} D = (1/3)Tr({{\textbf{D}}}^{tt}) = \frac{k_BT}{\eta _0} \frac{1}{3} Tr({{\textbf{M}}}^{*tt}) \end{aligned}$$

where *Tr*(...), the sum of the three diagonal components, is the same for every choice of the reference axes. The effective, orientationally averaged friction coefficient is, therefore,

57 $$\begin{aligned} f = \frac{k_BT}{D} = \frac{3\eta _0}{Tr({{\textbf{M}}}^{*tt})} \end{aligned}$$

This is, indeed, the *f* value that, according to Eq. (7), determines the sedimentation coefficient. Finally, we find that the equivalent radius for translational friction (the Stokes radius), as defined by Eq. (11), is

58 $$\begin{aligned} a_T \equiv R_{H,f} = [2 \pi {Tr({{\textbf{M}}}^{*tt})}]^{-1} \end{aligned}$$

For a spherical particle of radius *R*, the three diagonal components of ${{\textbf{M}}}^{*tt}$ are identical and equal to $1/(6 \pi \eta _0 R)$, and therefore, the equivalent radius is found to be *R* itself.

According to the fact enunciated above for the reduced mobility matrix, it is evident that *this equivalent radius depends just on the geometry of the solute particle and does not depend on any other quantity relative to the solute or the solvent*.

A similar conclusion can be demonstrated for hydrodynamic radii related to rotational diffusion and intrinsic viscosity. The formalism for these properties is more complex; the interested reader can find details elsewhere (García de la Torre and Carrasco 1998; García de la Torre et al. 2007; Carrasco and García de la Torre 1999a, b).
